# GWAS-Identified Variants for Obesity Do Not Influence the Risk of Developing Multiple Myeloma: A Population-Based Study and Meta-Analysis

**DOI:** 10.3390/ijms24076029

**Published:** 2023-03-23

**Authors:** José Manuel Sánchez-Maldonado, Antonio José Cabrera-Serrano, Subhayan Chattopadhyay, Daniele Campa, María del Pilar Garrido, Angelica Macauda, Rob Ter Horst, Andrés Jerez, Mihai G. Netea, Yang Li, Kari Hemminki, Federico Canzian, Asta Försti, Juan Sainz

**Affiliations:** 1 Genomic Oncology Area, GENYO, Centre for Genomics and Oncological Research, Pfizer/University of Granada/Andalusian Regional Government, PTS, 18016 Granada, Spain; josemanuel.sanchez@genyo.es (J.M.S.-M.); antonio.cabrera@genyo.es (A.J.C.-S.); 2 Division of Molecular Genetic Epidemiology, German Cancer Research Center (DKFZ), 69120 Heidelberg, Germany; subhayan.chattopadhyay@med.lu.se (S.C.); k.hemminki@dkfz-heidelberg.de (K.H.); 3 Division of Pediatric Neurooncology, German Cancer Research Center (DKFZ), German Cancer Consortium (DKTK), 69120 Heidelberg, Germany; a.foersti@kitz-heidelberg.de; 4 Hopp Children’s Cancer Center (KiTZ), 69120 Heidelberg, Germany; 5 Department of Biology, University of Pisa, 56126 Pisa, Italy; daniele.campa@unipi.it; 6 Hematology Department, Virgen de las Nieves University Hospital, 18012 Granada, Spain; mariap.garrido.sspa@juntadeandalucia.es; 7 Genomic Epidemiology Group, German Cancer Research Center (DKFZ), 69120 Heidelberg, Germany; angelicamacauda@gmail.com (A.M.); f.canzian@dkfz.de (F.C.); 8 Department of Internal Medicine and Radboud Center for Infectious Diseases, Radboud University Nijmegen Medical Center, 6525 GA Nijmegen, The Netherlands; rob.terhorst@radboudumc.nl (R.T.H.); mihai.netea@radboudumc.nl (M.G.N.); yang.li@helmholtz-hzi.de (Y.L.); 9 Department of Hematology, Experimental Hematology Unit, Vall d’Hebron Institute of Oncology (VHIO), University Hospital Vall d’Hebron, 08035 Barcelona, Spain; anjecayu@gmail.com; 10 Department for Immunology & Metabolism, Life and Medical Sciences Institute (LIMES), University of Bonn, 53115 Bonn, Germany; 11 Centre for Individualised Infection Medicine (CiiM) & TWINCORE, Joint Ventures between the Helmholtz-Centre for Infection Research (HZI) and the Hannover Medical School (MHH), 30625 Hannover, Germany; 12 Germany Division of Cancer Epidemiology, German Cancer Research Centre (DKFZ), 69120 Heidelberg, Germany; 13 Faculty of Medicine and Biomedical Center in Pilsen, Charles University in Prague, 30605 Pilsen, Czech Republic; 14 Department of Biochemistry and Molecular Biology I, University of Granada, 18071 Granada, Spain

**Keywords:** multiple myeloma, obesity, genetic variants, susceptibility

## Abstract

Multiple myeloma (MM) is an incurable disease characterized by the presence of malignant plasma cells in the bone marrow that secrete specific monoclonal immunoglobulins into the blood. Obesity has been associated with the risk of developing solid and hematological cancers, but its role as a risk factor for MM needs to be further explored. Here, we evaluated whether 32 genome-wide association study (GWAS)-identified variants for obesity were associated with the risk of MM in 4189 German subjects from the German Multiple Myeloma Group (GMMG) cohort (2121 MM cases and 2068 controls) and 1293 Spanish subjects (206 MM cases and 1087 controls). Results were then validated through meta-analysis with data from the UKBiobank (554 MM cases and 402,714 controls) and FinnGen cohorts (914 MM cases and 248,695 controls). Finally, we evaluated the correlation of these single nucleotide polymorphisms (SNPs) with cQTL data, serum inflammatory proteins, steroid hormones, and absolute numbers of blood-derived cell populations (*n =* 520). The meta-analysis of the four European cohorts showed no effect of obesity-related variants on the risk of developing MM. We only found a very modest association of the *POC5*_rs2112347G_ and *ADCY3*_rs11676272G_ alleles with MM risk that did not remain significant after correction for multiple testing (per-allele OR *=* 1.08, *p =* 0.0083 and per-allele OR *=* 1.06, *p =* 0.046). No correlation between these SNPs and functional data was found, which confirms that obesity-related variants do not influence MM risk.

## 1. Introduction

Multiple myeloma (MM) is an incurable disease characterized by the presence of malignant plasma cells in the bone marrow that secrete specific monoclonal immunoglobulins (also called M-protein) into the blood and/or urine [1,2]. M protein levels have been traditionally used to diagnose the disease and to monitor residual disease using protein electrophoresis (PEL), immunofixation electrophoresis (IFE), free light chain nephelometry (FLC), and liquid chromatography–mass spectrometry (LC-MS) methods [3]. However, despite the substantial advances during the last decade in identifying specific biomarkers and even the biological mechanisms underlying MM onset, there are no consistent risk factors other than male gender, age, African American ethnicity, obesity, and positive family history of lymphatohematopoietic cancer (LHC) and monoclonal gammopathy of undetermined significance (MGUS). Among these factors, obesity is a potentially interesting modifiable factor as it has been associated with several solid and hematological cancers [4,5,6,7,8,9,10,11], as well as tumor development by its interaction between cancer stem cells and macrophages or the tumor environment [12,13]. Although the analysis of both laboratory and clinical data has suggested complex associations between obesity and MM, the underlying genetic factors remain elusive. Thus, the aim of this study was to evaluate whether 32 GWAS-identified polymorphisms for obesity could influence the risk of MM. We also assessed whether these SNPs could exert their effect on MM risk by modulating host immune responses through comprehensive functional analysis.

## 2. Results

Selected polymorphisms did not deviate from Hardy Weinberg Equilibrium (HWE) in the control population (*p* < 0.001). The analysis of the discovery population only showed that each copy of the *MTCH2*_rs3817334T_ allele slightly decreased the risk of developing MM (OR *=* 0.89, *p =* 0.024). After the meta-analysis of all study cohorts, we could not replicate this finding but found a weak effect of the *ADCY3*_rs11676272_ and *POC5*_rs2112347_ SNPs on the risk of developing MM. However, these associations did not remain significant after multiple testing (OR *=* 1.06, *p =* 0.046 and OR *=* 1.08, *p =* 0.0083; Table 1). Association estimates did not substantially change after correction for body mass index (BMI).

**Figure 1:**
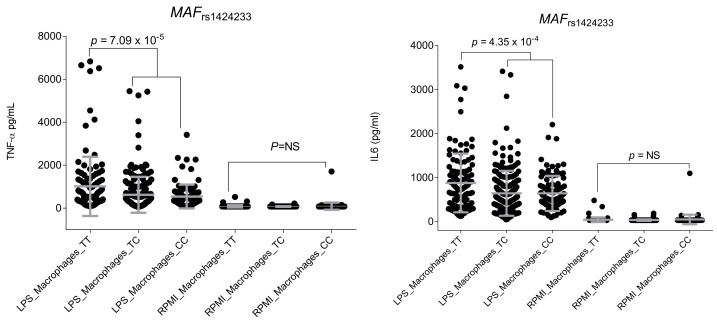
Correlation of the *MAF*_rs1424233_ SNP with TNFα and IL6 levels after stimulation of macrophages with LPS (*n =* 406).

Functional experiments did not suggest any functional effect of the *ADCY3*_rs11676272_ and *POC5*_rs2112347_ SNPs on the modulation of host immune responses, which suggested that, if any, the functional effect of these SNPs on MM risk was not mediated through the modulation of immune responses. Intriguingly, we found a novel and statistically significant association of the *MAF*_rs1424233_ SNP with levels of TNFα after the stimulation of human macrophages with LPS (*p =* 7.09 × 10^−5^; Figure 1). 

In addition, although it did not remain significant after correction for multiple testing, we found a correlation between this SNP and IL6 levels after stimulation of macrophages with LPS (*p =* 4.35 × 10^−4^; Figure 1), which might explain, at least in part, the link between obesity and inflammation. No significant correlation between the rest of the obesity-related SNPs and cQTL data, serum steroid hormone levels, serum inflammatory proteins, or absolute number of blood-derived cell populations was detected.

## 3. Discussion

This two-stage case–control association study showed that there is no significant association between GWAS-identified variants for obesity and MM risk. These findings were in agreement with a recent study that, using a Mendelian randomization strategy, demonstrated that SNPs associated with BMI, hip and waist circumference, waist-to-hip ratio, and childhood obesity were not involved in the modulation of MM risk [14]. In line with these negative findings, we neither found a positive correlation between obesity-related SNPs and cQTL data, serum inflammatory protein levels, steroid hormone levels, nor absolute numbers of blood-derived cell populations in the Human Functional Genomics Project (HFGP) cohort, which reinforced the hypothesis suggesting no effect of GWAS-identified SNPs for obesity in modulating MM risk. Interestingly, although it had no effect on MM risk, we found a potentially interesting correlation between the *MAF*_rs1424233_ polymorphism and TNFα and IL6 levels after stimulation of macrophages with LPS that confirmed the role of the *MAF* locus in modulating macrophage-mediated immune responses, a well-known phenomenon. MAF, which is expressed selectively in macrophages, positively regulates IL10 production in these cells after stimulation with LPS [15]. Similarly, it has been reported that *MAF* induces cytokine production [16] and promotes IL10-mediated anti-inflammatory responses through inhibition of the inflammasome [17]. Finally, another recent study showed that *MAF*, in addition to participating in the control of IL10 production and lipogenesis, is a negative regulator of IL2, which modulates Th1, Th2 and Th17 immune responses in a context-specific manner [18]. Considering that activated macrophages have the ability to initially produce proinflammatory cytokines (TNF, IL1β, IL6, and IL12) and, subsequently, induce the production of IL10 in response to LPS, it seems conceivable to suggest that genetic markers within the *MAF* locus might, at least in part, account for the link between obesity, lipogenesis and inflammation. 

## 4. Methods and Materials

### 4.1. Study Participants

This two-stage case–control association study included 658.359 subjects from four European cohorts. The discovery cohort consisted of 2121 MM cases and 2068 healthy controls recruited from four German clinical trials (GMMG-HD3/ISRCTN064413384, GMMG-HD4/ISRCTN64455289, GMMG-HD5/ISRCTN05745813, and GMMG-HD6/NCT02495922) [19] and a Spanish cohort that consisted of 1293 subjects (206 MM and 1087 healthy controls) recruited at two Spanish medical institutions (Virgen de las Nieves University Hospital, Granada, Spain, and Morales Meseguer Hospital, Murcia, Spain). Demographic and clinical details of the GMMG cohort have been previously published [19], and data regarding the Spanish cohort are included in Supplementary Table S1. The analysis of the discovery cohort was followed by meta-analysis with independent GWAS on 403,268 subjects from the UKBiobank (554 MM cases and 402,714 healthy controls; UKBiobank TOPMed-imputed) and 249,609 from the FinnGen cohort (914 MM cases and 248,695 healthy controls; Risteys7). Details on these GWAS data have been previously reported [20,21]. All MM patients were diagnosed according to the International Myeloma Working Group (IMWG) criteria [2,22,23]. The study was approved by the ethical committee of participant institutions, and all participants gave written informed consent to participate in the study.

### 4.2. SNP Selection and Genotyping

Genetic variants were selected on the basis of previously published research (Table 2) [24]. Genotyping of selected SNPs was carried out at GENYO (www.genyo.es; Granada, Spain) using KASPar assays (LGC Genomics, Hoddesdon, UK) according to previously reported protocols [25]. For internal quality control, ~5% of samples were randomly selected and included as duplicates. Concordance between the original and the duplicate samples for the selected SNPs was ≥99.0%. The call rate was higher than 90% with the exception of the *HIVEP1*_rs2228213_ SNP, which was removed from further analysis.

### 4.3. Statistical Analysis and Meta-Analysis

The HWE test was performed in the control group (alive subjects) by a standard observed-expected chi-square (χ^2^) test. Logistic regression analyses adjusted for age and gender were used to assess the effects of the genetic polymorphisms on MM risk in the discovery populations using a log-additive model. A gender-stratified association analysis adjusted for age was also performed to detect the gender-specific effects of selected SNPs on MM risk. All analyses were conducted using STATA (version 20.0). Subsequently, in order to validate the most interesting associations, a meta-analysis of the discovery populations with GWAS data of the UKBiobank and FinnGen cohorts was conducted using METAL. The I^2^ statistic was used to assess statistical heterogeneity between cohorts. The pooled OR was computed using a fixed-effect model. The Bonferroni method was used to account for multiple testing, and a *p*-value of 0.0016 (0.05/31 SNPs) was set as the study-wide significance threshold.

### 4.4. Cell Isolation, Differentiation, and Cytokine Quantitative Trait Loci (cQTL) in Relation to the GWAS-Identified Variants for Obesity

With the aim of determining whether those SNPs associated with obesity at the GWAS level had a role in modulating immune responses, we performed in vitro stimulation experiments and measured cytokine production (interferon (IFN) γ, interleukin (IL) 1Ra, IL1β, IL6, IL8, IL10, TNFα, IL17, and IL22) after stimulation of peripheral blood mononuclear cells (PBMCs), whole blood, or monocyte-derived macrophages (MDMs) from 408 healthy subjects of the 500 functional genomic (500FG) cohort from the HFGP with lipopolysaccharide (LPS; 1 or 100 ng/mL), phytohemagglutinin (PHA; 10 μg/mL), Pam3Cys (10 μg/mL), and CpG (ODN M362; 100 ng/mL) as an experimental model for cytokine production capacity. Details on PBMC isolation, macrophage differentiation and stimulation assays have been reported elsewhere [59,60]. The HFGP study was approved by the Arnhem-Nijmegen Ethical Committee (no. 42561.091.12), and biological specimens were collected after informed consent was obtained. After log transformation, cytokine levels were correlated with the SNPs of interest using a linear regression model with age and sex as co-factors in R (http://www.r-project.org/, accessed on 7 November 2022). A significance threshold of 0.000179 (0.05/31SNPs/9cytokines) was used for the cytokine quantitative trait loci (cQTL) analysis.

### 4.5. Correlation between GWAS-Identified Polymorphisms and Cell Counts of 91 Blood-Derived Immune Cell Populations and Serum/Plasmatic Proteomic Profile

We also investigated whether the selected polymorphisms had an impact on blood cell counts by analyzing a set of 91 manually annotated immune cell populations and genotype data from the 500FG cohort that consisted of 408 healthy subjects (Supplementary Table S2). Cell populations were measured by 10-color flow cytometry (Navios flow cytometer, Beckman Coulter) after blood sampling (2–3 h), and cell count analysis was performed using the Kaluza software (Beckman Coulter, v.1.3). In order to reduce inter-experimental noise and increase statistical power, cell count analysis was performed by calculating parental and grandparental percentages, which were defined as the percentage of a certain cell type within the cell-populations one or two levels higher in the hierarchical definitions of cell sub-populations [61]. Detailed laboratory protocols for cell isolation, reagents, gating and flow cytometry analysis have been reported elsewhere [62], and the accession number for the raw flow cytometry data and analysed data files are available upon request to the authors (http://hfgp.bbmri.nl). A proteomic analysis was also performed in serum and plasma samples from the 500FG cohort. Circulating proteins were measured using the commercially available Olink Inflammation panel (Olink, Sweden), which resulted in the measurement of 103 different biomarkers (Supplementary Table S3). Protein levels were expressed on a log2-scale as normalized protein expression values and normalized using bridging samples to correct for batch variation. Considering the number of proteins (*n =* 103) and cell populations (*n =* 91) tested, *p*-values of 0.000016 and 0.000017 were, respectively, set as the significant thresholds for the proteomic and cell-level variation analyses.

### 4.6. Correlation between Obesity-Related SNPs and Serum Steroid Hormones

Finally, we also measured serum levels of seven steroid hormones (androstenedione, cortisol, 11-deoxy-cortisol, 17-hydroxy progesterone, progesterone, testosterone, and 25 hydroxy vitamin D3) in 280 healthy subjects from the 500FG cohort that did not undergo hormone replacement therapy or take oral contraceptives. After log transformation, the correlation between steroid hormone levels and obesity SNPs was evaluated by linear regression analysis adjusted for age and sex. The significance threshold was set to 0.00023 (0.05/31 SNPs/7 hormones). Complete protocol details of steroid hormone measurements have been reported elsewhere [59].

## 5. Conclusions

In summary, our findings suggest that GWAS-identified variants for obesity do not influence the risk of MM and that the *MAF* locus might play a role in modulating the inflammatory alterations and lipogenesis, leading to the development of obesity.

## Figures and Tables

**Table 1:** Meta-analysis of the discovery cohort with GWAS data from the UKBiobank and FinnGen projects.

Gene_SNP ID	Discovery Cohort (*n =* 5482)		UKBiobank (*n =* 403,268)		FinnGen (*n =* 249,609)		Meta-Analysis (*n =* 658,359)		
	OR (95% CI) ^∂^	*p*	OR (95% CI)	*p*	OR (95% CI)	*p*	OR (95% CI)	*p*	*P_Het_*
*ADCY3_*rs11676272	1.04 (1.13–0.95)	0.36	1.05 (0.93–1.17)	0.44	1.09 (0.99–1.19)	0.084	**1.06 (1.00–1.11)**	**0.046**	0.47 0.36
*ADPGK_*rs7164727	1.01 (1.10–0.91)	0.86	0.98 (0.87–1.10)	0.71	1.07 (0.97–1.18)	0.20	0.98 (0.92–1.04)	0.63	
*AKAP6_*rs17522122	0.94 (1.06–0.84)	0.31	1.08 (0.96–1.21)	0.22	1.02 (0.93–1.12)	0.66	1.01 (0.95–1.08)	0.70	0.22 0.97
*BDNF_*rs6265	1.00 (1.36–0.74)	1.00	0.97 (0.83–1.12)	0.65	0.97 (0.86–1.10)	0.63	0.97 (0.88–1.07)	0.51	
*DNASE1_*rs1053874	1.08 (1.20–0.96)	0.19	1.04 (0.92–1.18)	0.52	0.91 (0.82–1.00)	0.056	1.00 (0.93–1.06)	0.88	0.12 0.73
*FAIM2_*rs7138803	0.99 (1.08–0.91)	0.85	1.01 (0.90–1.13)	0.90	0.95 (0.86–1.04)	0.28	0.98 (0.93–1.04)	0.48	
*FLT3_*rs1933437	0.94 (1.04–0.83)	0.27	0.98 (0.85–1.09)	0.69	0.91 (0.81–1.01)	0.090	0.94 (0.87–1.00)	0.054	0.77 0.34 **0.008**
*FTO_*rs1421085	0.95 (1.04–0.85)	0.33	1.10 (0.97–1.24)	0.14	1.01 (0.93–1.11)	0.77	1.01 (0.95–1.06)	0.78	
*FTO_*rs7190492	1.06 (1.16–0.98)	0.15	0.93 (0.79–1.05)	0.27	1.01 (0.92–1.10)	0.79	1.02 (0.96–1.08)	0.54	
*GNPDA2_*rs10938397	1.05 (1.14–0.96)	0.26	1.01 (0.90–1.12)	0.93	1.01 (0.93–1.10)	0.80	1.03 (0.97–1.08)	0.37	0.62 0.69
*GPRC5B_*rs12444979	1.13 (1.62–0.79)	0.51	0.96 (0.82–1.13)	0.65	1.03 (0.90–1.17)	0.70	1.01 (0.91–1.12)	0.85	
*ITH4_*rs4687657	1.04 (1.15–0.94)	0.48	0.97 (0.85–1.10)	0.61	1.07 (0.96–1.18)	0.22	1.03 (0.97–1.10)	0.33	0.72 0.93
*KCTD15_*rs11084753	1.04 (1.17–0.92)	0.53	0.99 (0.87–1.10)	0.91	0.99 (0.89–1.08)	0.84	1.00 (0.94–1.07)	0.88	
*LMOD1_*rs2820312	0.99 (1.09–0.91)	0.89	0.96 (0.85–1.08)	0.49	0.96 (0.87–1.06)	0.40	0.97 (0.92–1.03)	0.36	0.94 0.61 0.99
*LOC400652_*rs17782313	1.06 (1.15–0.96)	0.23	1.01 (0.89–1.15)	0.88	1.03 (0.92–1.16)	0.57	1.04 (0.97–1.10)	0.24	
*MAF_*rs1424233	0.99 (1.07–0.92)	0.83	1.01 (0.89–1.11)	0.89	1.00 (0.91–1.08)	0.99	1.00 (0.95–1.05)	0.95	
*MC4R_*rs17700633	1.04 (1.15–0.94)	0.43	0.99 (0.88–1.12)	0.92	0.91 (0.82–1.02)	0.096	0.98 (0.92–1.05)	0.60	0.37 0.92 0.43
*MST1R_*rs2230590	0.95 (1.04–0.85)	0.26	1.00 (0.88–1.14)	0.98	0.97 (0.89–1.07)	0.57	0.97 (0.91–1.03)	0.30	
*MTCH2_*rs3817334	**0.89 (0.99–0.81)**	**0.024**	0.98 (0.88–1.10)	0.78	0.99 (0.91–1.08)	0.91	0.95 (0.90–1.01)	0.12	
*NEGR1_*rs2815752	0.95 (1.04–0.86)	0.32	1.03 (0.91–1.14)	0.63	1.05 (0.96–1.14)	0.29	1.01 (0.95–1.06)	0.83	0.16
*NPC1_*rs1805081	1.16 (1.35–0.91)	0.19	0.93 (0.83–1.05)	0.24	1.02 (0.93–1.11)	0.71	1.00 (0.93–1.07)	0.96	0.20 0.88
*NT5C2_*rs11191580	0.98 (1.14–0.79)	0.81	0.97 (0.78–1.19)	0.77	1.03 (0.88–1.21)	0.73	1.00 (0.89–1.09)	0.94	
*PCSK1_*rs6235	1.01 (1.11–0.91)	0.92	1.10 (0.98–1.21)	0.099	1.05 (0.95–1.14)	0.35	1.05 (0.98–1.12)	0.16	0.36
*POC5_*rs2112347	1.09 (1.17–1.00)	0.055	1.07 (0.95–1.21)	0.28	1.07 (0.98–1.18)	0.13	**1.08 (1.02–1.13)**	**0.0083**	0.96
*SEC16B_*rs543874	1.03 (1.14–0.90)	0.63	0.93 (0.81–1.08)	0.35	1.04 (0.92–1.16)	0.56	1.01 (0.93–1.08)	0.87	0.67
*SH2B1_*rs7359397	0.94 (1.06–0.84)	0.33	0.97 (0.86–1.09)	0.63	**1.12 (1.02–1.23)**	**0.017**	1.03 (0.96–1.09)	0.41	**0.043** 0.19
*STK33_*rs10769908	1.00 (1.10–0.88)	0.96	0.91 (0.77–1.03)	0.14	1.06 (0.97–1.15)	0.17	1.00 (0.94–1.06)	0.91	
*TFAP2B_*rs2206277	1.03 (1.16–0.91)	0.65	1.13 (0.96–1.32)	0.14	1.00 (0.90–1.10)	0.97	1.04 (0.96–1.11)	0.34	0.17
*TMEM18_*rs6548238	0.94 (1.06–0.83)	0.30	1.05 (0.89–1.18)	0.53	0.99 (0.86–1.10)	0.84	0.98 (0.91–1.06)	0.65	0.73
*TRAF3_*rs10133111	1.05 (1.19–0.92)	0.46	1.00 (0.87–1.16)	0.97	1.10 (0.99–1.23)	0.080	1.06 (0.99–1.15)	0.11	0.68
*UHRF1BP1_*rs11755393	0.99 (1.14–0.82)	0.90	1.02 (0.90–1.14)	0.79	0.97 (0.88–1.07)	0.53	0.99 (0.91–1.06)	0.72	0.95
*ZZZ3_*rs17381664	0.97 (1.06–0.87)	0.51	0.95 (0.84–1.07)	0.39	–	–	0.96 (0.88–1.03)	0.29	0.91

Abbreviatures: SNP, single-nucleotide polymorphism; OR, odds ratio; CI, confidence interval. A fixed effect model was assumed for the meta-analysis of all cohorts. ^∂^ Association estimates were adjusted for age and sex and were calculated according to log-additive model of inheritance. *p* ≤ 0.05 in bold.

**Table 2:** Selected obesity-related SNPs.

Gene Name	dbSNP rs#	Effect Allele	Context	References
*ADCY3*	rs11676272	G	missense_variant	[26,27,28,29,30,31]
*AKAP6|NPAS3*	rs17522122	G	3_prime_UTR_variant	[32,33,34,35,36]
*ADPGK* *|ADPGK-AS*	rs7164727	T	downstream_gene_variant	[32,34,35,37]
*BDNF|BDNF-AS*	rs6265	A	missense_variant	[29,33,35,38,39,40,41]
*DNASE1*	rs1053874	A	missense_variant	[37,42]
*FAIM2|BCDIN3D*	rs7138803	A	intergenic_variant	[27,29,32,33,34,35,36,37,38,43,44,45,46]
*FLT3*	rs1933437	C	missense_variant	[35]
*FTO*	rs1421085	C	intron_variant	[38]
*FTO*	rs7190492	A	intron_variant	[40]
*GNPDA2*	rs10938397	G	intergenic_variant	[27,29,32,33,34,35,36,37,38,43,44,45,47,48,49,50,51,52]
*GPRC5B|GPR139|PDILT*	rs12444979	T	intergenic_variant	[44]
*HIVEP1*	rs2228213	A	missense_variant	[33,34,35,38]
*ITH4*	rs4687657	T	missense_variant	[37]
*KCTD15*	rs11084753	A	intergenic_variant	[51]
*LMOD1*	rs2820312	A	missense_variant	[34,37,42,51]
*LOC400652|LOC342784*	rs17782313	C	intergenic_variant	[32,51,53,54]
*MAF*	rs1424233	T	regulatory_region_variant	[54]
*MC4R*	rs17700633	A	n/s	[53]
*MST1R*	rs2230590	C	missense_variant	[36,37,42]
*MTCH2*	rs3817334	T	intron_variant	[32,34,35,36,37,38,44,45]
*NEGR1|LOC105378797*	rs2815752	C	intron_variant	[44,51]
*NPC1|SLC35F4*	rs1805081	C	missense_variant	[54]
*NT5C2*	rs11191580	C	intron_variant	[36]
*PCSK1*	rs6235	C	missense_variant	[33]
*POC5|FLJ35779*	rs2112347	G	intergenic_variant	[29,32,33,34,35,36,37,38,43,44,45,48,50]
*SEC16B*	rs543874	G	upstream_gene_variant	[26,27,29,32,33,34,35,36,37,38,44,45,47,49,50,55,56,57]
*SH2B1*	rs7359397	T	intron_variant	[44]
*STK33*	rs10769908	C	intron_variant	[51]
*TFAP2B*	rs2206277	A	intron_variant	[29,33,36,38,43,58]
*TMEM18*	rs6548238	T	TF_binding_site_variant	[51]
*TRAF3*	rs10133111	A	3_prime_UTR_variant	[36,37]
*UHRF1BP1*	rs11755393	G	missense_variant	[37,42]
*ZZZ3*	rs17381664	C	intron_variant	[32,35,36,43]

Abbreviature: SNP, single nucleotide polymorphism.

## Data Availability

Genetic data from the Spanish cohort are available at the GENYO data infrastructure (https://ftp.genyo.es/, accessed on 20 March 2023). These data are available upon reasonable request. Data from the German population are available from four German clinical trials (GMMG-HD3/ISRCTN064413384, GMMG-HD4/ISRCTN64455289, GMMG-HD5/ISRCTN05745813 and GMMG-HD6/NCT02495922). Functional data used in this study are available at the BBMRI-NL data infrastructure (https:// hfgp.bbmri.nl/, accessed on 2 July 2021), where they have been meticulously catalogued and archived using the MOLGENIS open-source platform for scientific data. This allows flexible data querying and downloading, including sufficiently rich metadata and interfaces for machine processing (R statistics, REST API) using FAIR principles to optimize Findability, Accessibility, Interoperability, and Reusability. These datasets are not publicly available because they contain information that could compromise the research participants’ privacy.

## Supplementary Materials

The supporting information can be downloaded at: https://www.mdpi.com/article/10.3390/ijms24076029/s1.
