# Supplementary material for: GWAS-Identified Variants for Obesity Do Not Influence the Risk of Developing Multiple Myeloma: A Population-Based Study and Meta-Analysis
Source: Int J Mol Sci. 2023 Mar 23;24(7):6029. doi: 10.3390/ijms24076029 (PMC10094344; doi:10.3390/ijms24076029)
Supplement: Supplementary file 1 [file ijms-24-06029-s001.zip › Sanchez-Maldonado_JM_[1]. Supplementary_Table_S1.docx]

**Table S1.** Demographic and clinical characteristics of MM patients and healthy controls from Spanish Cohort.

| **Demographic characteristics** | Spanish Cohort  (n=1293) | |
| --- | --- | --- |
|  | MM cases  (n=206) | Healthy Controls  (n=1087) |
|  |  |  |
| *Age (years, average*±SD*)* | 63.98 ± 11.77 | 52.49 ± 6.54 |
| *Sex ratio (female/male)* | 0.93 (99/107) | 0.81 (488/601) |
|  |  |  |
| **Disease stage** |  |  |
|  |  |  |
| ***Durie-Salmon*** |  |  |
| *1A/1B* | 21 (10.20) | - |
| *2A/2B* | 55 (26.70) | - |
| *3A/3B* | 124 (60.19) | - |
| *Unknown* | 6 (02.91) |  |
|  |  |  |
| ***ISS Stage*** |  |  |
| *Stage I* | 56 (27.18) | - |
| *Stage II* | 75 (38.27) | - |
| *Stage III* | 65 (31.55) | - |
| *Unknown* | 10 (04.85) |  |
|  |  |  |

Age is represented as means ± standard. ISS (International staging system) and Durie-Salmon scores as n (%).
